# Investigation and Stability Assessment of Three Sill Pillar Recovery Schemes in a Hard Rock Mine

**DOI:** 10.3390/en15103797

**Published:** 2022-05-21

**Authors:** Huawei Xu, Derek B. Apel, Jun Wang, Chong Wei, Krzysztof Skrzypkowski

**Affiliations:** 1 School of Mining and Petroleum Engineering, University of Alberta, Edmonton, AB T6G 2R3, Canada; hx1@ualberta.ca (H.X.); jun8@ualberta.ca (J.W.); cwei4@ualberta.ca (C.W.); 2 Faculty of Civil Engineering and Resource Management, AGH University of Science and Technology, 30-059 Kraków, Poland

**Keywords:** hard rock mine, sill pillar recovery, upper bench level, ground settlement, tangential stress criteria, burst potential index (BPI)

## Abstract

In Canada, many mines have adopted the sublevel stoping method, such a blasthole stoping (BHS), to extract steeply deposited minerals. Sill pillars are usually kept in place in this mining method to support the weight of the overburden in underground mining. To prolong the mine’s life, sill pillars will be recovered, and sill pillar recovery could cause failures, fatality, and equipment loss in the stopes. In this paper, three sill pillar recovery schemes—SBS, SS1, and SS2—were proposed and conducted to assess the feasibility of recovering two sill pillars in a hard rock mine by developing a full-sized three-dimensional (3D) analysis model employing the finite element method (FEM). The numerical model was calibrated by comparing the model computed ground settlement with the in situ monitored ground settlement data. The rockburst tendency of the stope accesses caused by the sill pillar recovery was assessed by employing the tangential stress (Ts) criterion and burst potential index (BPI) criterion. All three proposed sill pillar recovery schemes were feasible and safe to recover the sill pillars in this hard rock mine, and the scheme SBS was the optimum one among the three schemes.

## 1. Introduction

In underground mining, to improve production, several mining levels are generally active in the mining process at different mining depths, simultaneously. The mining process will cause redistributed stresses, which may transfer horizontally and vertically. The transferred stress may contribute to failure of the stopes and damage to the mining equipment. For the sake of mining zone safety, sill pillars are commonly reserved to prevent the transfer of the redistributed stress, especially in the steeply dipping orebodies. In most cases, sill pillars are recovered to prolong the mine life and maximize the usage of minerals. Pillar recovery is the practice of developing several pillars and then extracting the pillars, and it is considered the most hazardous form of underground mining [1,2]. It can trigger risks during pillar recoveries such as overlaying rock breakage, stope failure, and pillar failure [1,2,3,4].

To better assess the stability and improve the safety of miners and mining equipment during sill pillar recovery, scholars initiated and proposed empirical, analytical theories, and numerical modelling methods. To understand the mechanisms of pillar failures, Hudyma and Potvin [5,6] studied the conventional ground control instruments, in situ field visual observations, and numerical analysis modelling. Mark [1,7,8,9,10,11,12,13] and Iannacchione [14] assessed the major hazard risks and analyzed the MHRA techniques to evaluate sill pillar recovery in the room-and-pillar mining method. Zur [15] proposed the enhanced cemented rockfill to recover pillars and revealed that the pillars used the passive confinement effect of cemented rockfill (CRF) to increase the post-peak load-bearing ability. Zhukova [16] employed the monitored underground seismic registrations and proposed mathematical models to improve the safety operations in pillar recovery. Langston [17] designed the stope layout, pillar extraction schedule, and ground support to recover sill pillars safely. Sainsbury [18,19,20] examined sill pillar stability and failure mechanisms and simulated pillar recovery by replacing the orebodies with laboratory-tested stabilized rockfill to solve the technical risks caused by the proposed extraction method. Ghasemi [21,22] assessed the risk of pillar recovery operations and classified that risk into four categories by using failure indicators. Beruar [23] and Valley [24] developed and optimized the mining sequence and suggested new directions for the different methods and potential shortcomings to avoid during pillar recovery. Townend [25] initiated five mitigation strategies to mitigate the high-stress concentration while mining sill pillars. Zhou [26] investigated the instability of large mined-out areas triggered by dynamic disturbance resulting from residual pillar recovery. Kyei [27] selected the suitable blasted rock particle size for making CRF to backfill the mined-out stopes for sill pillar recovery.

Cemented rockfill (CRF) is widely used to improve the stability and safety of the whole mining pipe and the sill pillar recovery, while, in many cases, compared with the host rocks, the strength of the CRF is relatively lower [18,19,20,28,29,30,31,32,33]. Sill pillar recovery with the influence of backfilled CRF in the blasthole stoping (BHS) mining method has not been widely discussed. In this paper, three schemes of sill pillar recovery were proposed and implemented to investigate and assess the feasibility of recovering the two sill pillars in a hard rock mine by applying the finite element method (FEM). 

## 2. Background and Methodology

**Figure 1:**
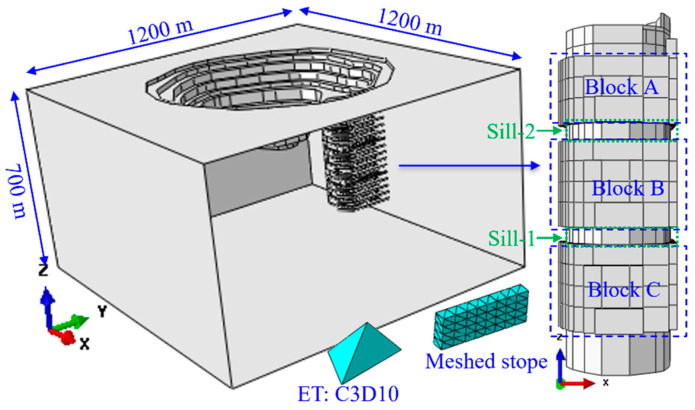
Two sill pillars in pipe MP#1.

The hard rock mine in this study initially operated as an open pit mine. Once the open-pit mining was completed, the mining operation shifted to the underground. Due to the geological structure and deposition of the minerals, the blasthole stoping (BHS) mining method was used to excavate the steeply dipped mining pipe. According to the mining plan, there are three planned mining blocks in mining pipe MP#1, namely Block-A, Block-B, and Block-C, as shown in Figure 1. There are two initially kept sill pillars between the mining blocks—Sill-1 and Sill-2. The two sill pillars work as the protective pillars, which can effectively prevent the transfer of redistributed stress from one block to another [30]. After the completion of the excavation of three mining blocks, the feasibility of recovery of these two sills then plays a significant role in the resource development plan and prolonging the mine’s life.

Full-sized 3D models can estimate the deformations of underground openings and explore the mining-induced stress redistribution paths better than 2D analysis models [30,34,35,36,37]. Therefore, to better represent the complicated geometry of the stopes and haulages in the mining pipe at the hard rock mine, a full-sized elastoplastic three-dimensional (3D) model was developed by employing ABAQUS codes [38]. As illustrated in Figure 1, to better simulate the stress change paths in the stopes and haulages, the ten-node quadratic tetrahedron mesh element type (ET: C3D10) was used to mesh the stopes and analysis domain to conduct the simulation [38]. To make precise predictions, the model was created based on the real geometry of the stopes and haulages at the mine, and some modifications were made. To eliminate the influence of boundary effects of the model, an optimization study of the size of the analysis domain and a mesh convergence were conducted, and the analysis domain has a size of 1200 m × 1200 m × 700 m (length × width × depth), as shown in Figure 1 [39]. At the bottom of the model, the boundary conditions are applied to fix the bottom, and the top surface is set free. In addition, the horizontal restraints on both X and Y directions are applied to the four vertical boundaries of the model. 

To achieve the parameters of the rock samples, several laboratory tests were conducted. Table 1 shows the rock mechanical properties applied in the model in this study [39]. Here, *γ* is the unit weight, *C* is the cohesive strength, *ϕ* is the angle of friction, *E* is the elastic Young’s modulus, *ν* is the Poisson’s ratio, and *σ_c_* is the uniaxial compressive strength. 

## 3. Calibration and Verification of the Finite Element (FE) Model

The finite element (FE) numerical analysis model is capable to conduct general study and helping the engineer to understand better the “real world” of the underground space [40,41,42,43,44,45]. Model calibration is a vital step in checking the reliability of the developed numerical model by comparing the monitored and recorded in situ data with the computed data from the developed numerical model.

**Figure 2:**
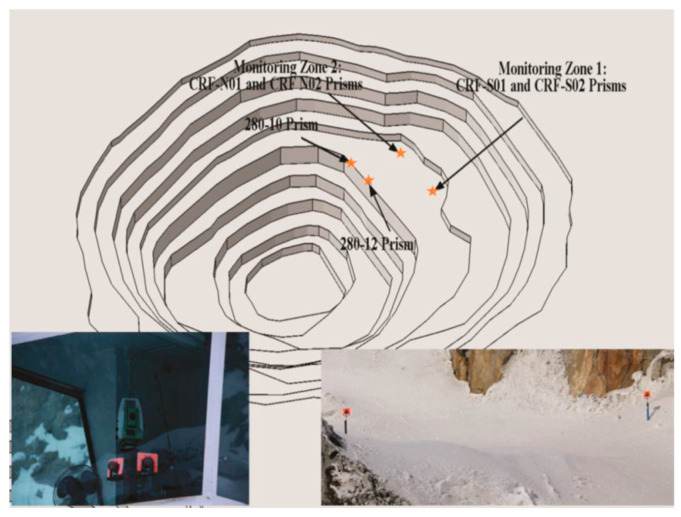
The open pit bench at the mine [30].

In this paper, to calibrate the developed FE model, several monitor prisms were installed on the open pit benches to measure the ground surface subsidence induced by the mining activities in the mining pipe. Figure 2 presents the location of the installed monitor prisms on the benches. Monitoring zone 1 has two prisms: CRF-S01 and CRF-S02. Monitoring zone 2 has two prisms: CRF-N01 and CRF-N02. Both monitoring zone 1 and monitoring zone 2 are on the boundary of the top surface of the mining pipe MP#1. Monitoring zone 3 also has two prisms: 280-10 and 280-12.

Before implementing the simulation of the recovery process of the sill pillars, the developed numerical model was calibrated by comparing the ground surface settlement at monitoring zone 1 (prism CRF-S01, CRF-S02) and monitoring zone 2 (prism CRF-N01, CRF-N02) with the computed ground surface settlement from the numerical model. As a result, it takes 83 simulation steps, based on in situ production schedules, to excavate and backfill the three mining blocks in the developed analysis model.

**Figure 3:**
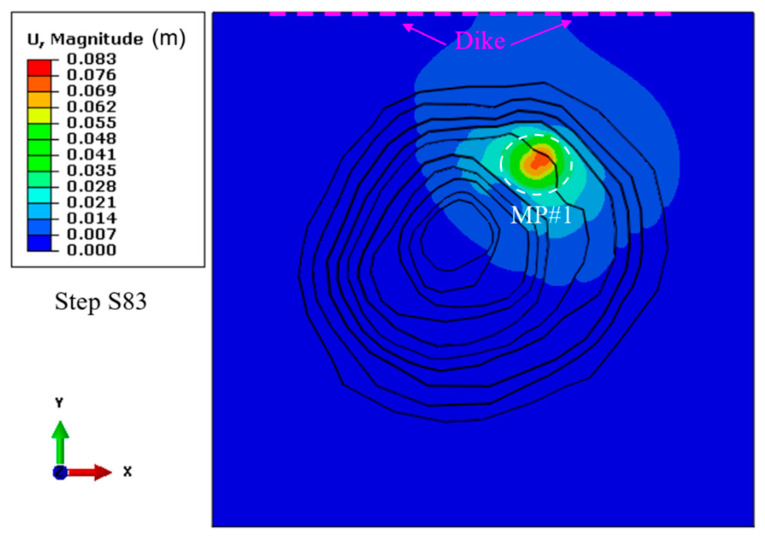
Computed displacement in the FE model before sill pillar recovery.

The profile of the computed displacement of the open pit slopes in the FE model is shown in Figure 3. The main areas with maximum ground settlement are over the mining pipe MP#1, where the mining activities are in active production. Though the three mining blocks were backfilled with cemented rockfill (CRF), the open pit boundary in the developed model, where the dike is located, still sees a tiny increase in displacement after the completion of the excavation and backfilling of the three mining blocks. The stability and safety of the protective dike play a significant role in mine site safety.

The comparison of the accumulated ground displacement between the in situ recorded data and the computed FE model results is shown in Table 2. The relative errors in different zones are 2.57% and 8.01% in zone 1 and 2, respectively. The average relative error is 5.29%, which is acceptable in mining engineering simulation analysis [30,46,47,48]. 

The analysis model calibration conducted via comparison of the ground displacement caused by the excavation and backfilling process of the three mining blocks, between the recorded in situ data and the computed simulation results of the FE model verifies that the developed FE analysis model is reliable and capable of conducting and implementing the simulation steps of the sill pillar recovery process.

## 4. Schemes of Sill Pillar Recovery

From the above FE model calibration, by comparison of the in situ recorded displacement and the computed results in the developed FE model, the developed FE model is capable and precise in conducting the numerical simulation to assess the feasibility of the recovery of the two sill pillars in the mining pipe MP#1.

### 4.1. Primary and Secondary Mining Sequence in Blasthole Stoping Mining Method

**Figure 4:**
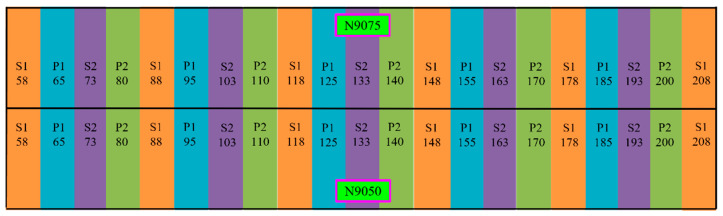
The stope mining sequence in the example two levels.

All three sill pillar recovery schemes follow the primary and secondary mining sequence to keep the sequence constant with the three mining blocks’ mining schedules. The primary sequence stopes will be excavated firstly, and then the secondary sequence stopes will be excavated. Figure 4 shows an example configuration of the mining stopes of two mining levels. The colors orange, blue, purple, and green represent the mining sequence. Blue and green represent the primary mining sequence, and orange with purple for the second mining sequence. Here, P stands for the primary sequence, P1 means those stopes will be mined first in the primary sequence, and P2 stands for the second round of mined stopes in the primary sequence. Then, S stands for the secondary sequence, S1 means the first round of mined stopes in the secondary sequence, and S2 means the second round of mined stopes in the secondary sequence. When the stoping starts, stope P1-65 will first be mined out, followed by P1-95. After the first round in the primary sequence finishes, the second round of the primary sequence starts, and then the first round in the secondary sequence starts, followed by the second round in the secondary sequence. The stope of S2-193 will be the last mined-out stope in this level. Each stope will be backfilled as soon as it is mined.

### 4.2. Three Proposed Sill Pillar Recovery Schemes

**Figure 5:**
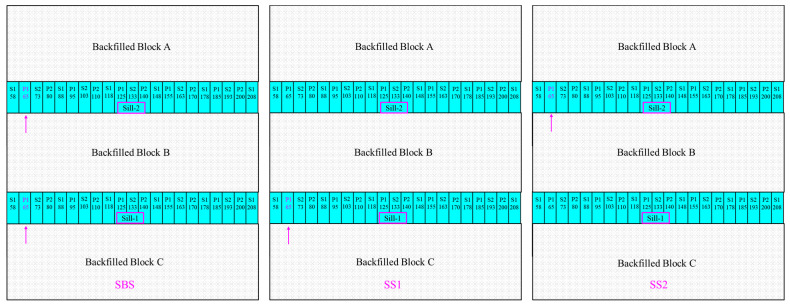
Profile of the sill pillar recovery scheme of SBS, SS1, and SS2.

Three sill pillar recovery schemes are proposed to recover the two sill pillars. To keep the mining sequence consistent with the designed sequence schedule at the mine, the mining sequence to recover the two sill pillars follows the sequence of mining and backfilled the three mining blocks in mining pipe MP#1. The first mined stopes in the three recovery schemes of the two sill pillars are labelled with the color magenta, as shown in Figure 5. 

The first proposed scheme of the recovery process starts at both sill pillars (SBS), from the stope P1-65 to the two sill pillars are excavated and backfilled, and the simulation steps start from step S84 to step S94. The second proposed scheme of the recovery process starts at Sill-1 (SS1), as shown in Figure 5, from the stope P1-65, and when all the stopes are excavated in Sill-1, then the mining process moves to Sill-2, until the two sill pillars are recovered and backfilled, and then simulation steps start from step S84 to step S98. Finally, the third proposed scheme of recovery process starts at Sill-2 (SS2), as shown in Figure 5, from the stope P1-65, and when all the stopes in Sill-2 are excavated, the mining process moves to Sill-1, until all the sill pillars are recovered and backfilled, and similarly with SS1, the simulation steps start from step S84 to step S98.

## 5. Displacement Caused by the Three Sill Pillar Recovery Schemes

### 5.1. Displacement at the Three Monitoring Zones

When conducting the recovery of the two sill pillars, the open pit benches are still in function to transport the orebodies from the sill pillars in mining pipe MP#1 to the ground surface. Then, the benches’ stability is still a key issue to be considered. 

**Figure 6:**
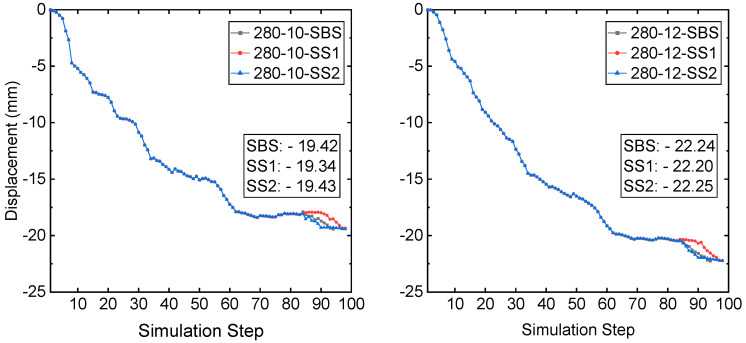
A comparison of displacement at monitored prism 280-10 and 280-12.

From simulation step S84, the recovery process starts. Scheme SBS takes 11 simulation steps to recover and backfill the two sill pillars, and the simulation step ends at step S94. Both schemes SS1 and SS2 take 15 steps to recover and backfill the two sill pillars, and the simulation step ends at step S98. Though it takes different simulation steps to recover and backfill the two sill pillars, the mining-induced displacements at the monitored zones during the recovery process are almost the same. Figure 6 presents the computed displacement from the FE model at prisms 280-10 and 280-12. The final three displacements at prism 280-10 are −19.42 mm, −19.34 mm, and −19.43 mm, from recovery schemes SBS, SS1, and SS2, respectively. For the prism 280-12, the final three displacements are −22.24 mm, −22.20 mm, and −22.25 mm, respectively, from recovery schemes SBS, SS1, and SS2.

**Figure 7:**
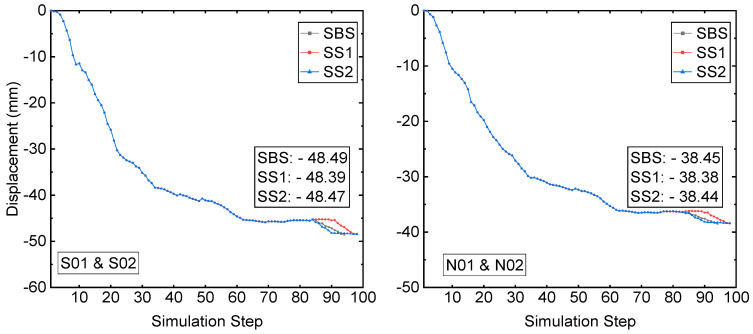
A comparison of the displacement at monitored sites S01-02 and N01-02.

The displacements at monitoring zone 1 and zone 2 are shown in Figure 7. Like the displacement at monitoring zone 3, the final displacements of three different recovery schemes are very close at both monitoring zone 1 and zone 2. At the prism CRF-S, the three displacements are −48.49 mm, −48.39 mm, and −48.47 mm, respectively, from recovery schemes SBS, SS1, and SS2. The three displacements at the monitoring prism CRF-N are −38.45 mm, −38.38 mm, and −38.44 mm, from recovery schemes SBS, SS1, and SS2, respectively. 

During the mining and backfilling of the three mining blocks, the mining-induced displacement at the three monitoring zones reaches a value of 46.7 mm (CRF-S) and 39.55 mm (CRF-N), respectively. From the mining-induced displacement view, the three different schemes of sill pillar recovery have very close results at the two monitoring zones. Also, the displacement induced by the process of sill pillar recovery sees a slight increase at all three monitoring zones. Table 3 presents the displacement induced by the process of sill pillar recovery at the monitoring prisms.

All displacements induced by the three sill pillar recovery schemes are not more than 3 mm. Therefore, the backfilled CRF in the three mining blocks provides sufficient support for the process of sill pillar recovery. Furthermore, all three schemes of sill pillar recovery can be implemented.

### 5.2. Displacement of the Upper Levels 

**Figure 8:**
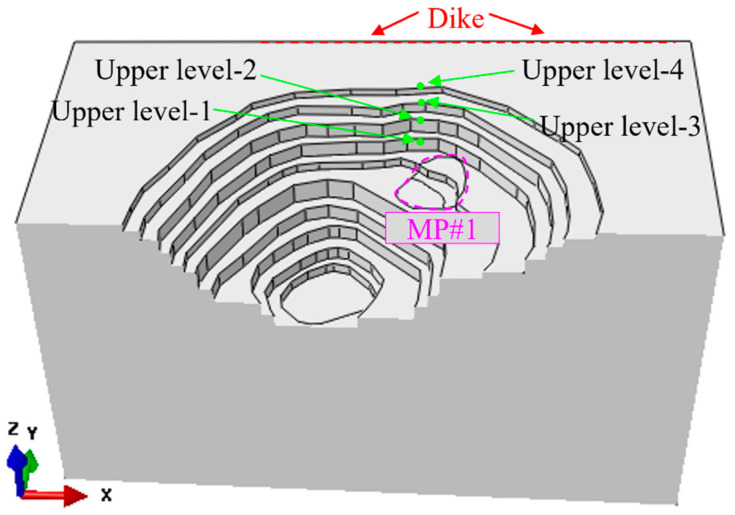
The chosen monitored location on the upper bench levels.

From the upper levels to the MP#1 mining pipe, there are four levels of open-pit benches, as shown in Figure 8. Several locations are chosen on bench levels in the numerical FE model to monitor the displacement on the benches caused by the mining activities and sill pillar recovery process in the mining pipe MP#1.

The four selected upper levels are marked with upper level-1 (UL-1), upper level-2 (UL-2), upper level-3 (UL-3), and upper level-4 (UL-4), as shown in Figure 8. Here, upper level-4 (UL-4) is the ground surface at the mine. The elevation between the top surface of mining pipe MP#1 and the ground surface is 94 m. Therefore, the depths of the four bench levels are 0 m, −14 m, −34 m, and −64 m, from upper level-4 (UL-4) to upper level-1 (UL-1), from the ground surface to the top surface of the bench level-1 (UL-1).

**Figure 9:**
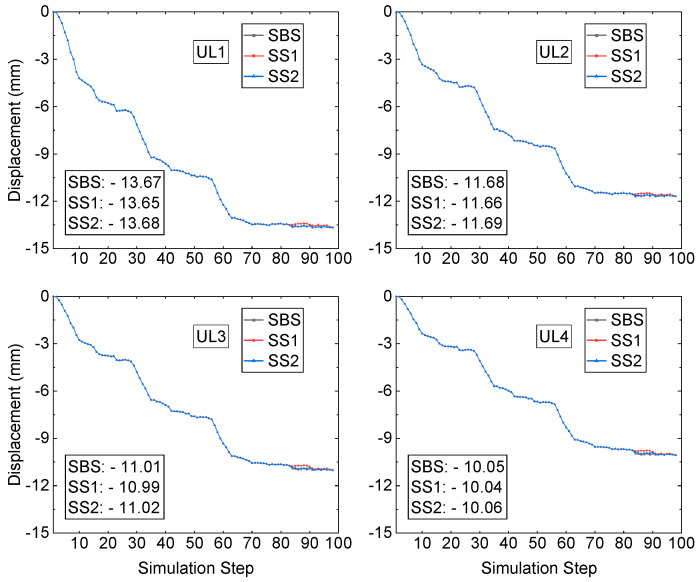
A displacement comparison at the monitored locations.

The average displacements at the analyzed locations on the four upper bench levels in the numerical model caused by the mining activities and the three sill pillar recovery schemes process are presented in Figure 9. From the beginning of the mining production in mining pipe MP#1 to the state of three mining blocks which were all mined and backfilled, the process of mining and backfilling in pipe MP#1 caused the displacement of −13.50 mm, −11.56 mm, −10.73 mm, and −9.76 mm, from upper bench level-1 (UL-1) to upper bench level-4 (UL-4). Compared to the displacement of the monitored prisms at the monitoring zones around the boundary of the top surface of mining pipe MP#1, the displacements at the upper bench levels caused by the mining activities of three mining blocks in mining pipe MP#1 are much smaller. From upper bench level-1 (UL-1) to upper bench level-4 (UL-4), the mining-induced ground displacement at the bench level decreased constantly, which proves the influence on the displacement of the upper benches caused by the mining activities in mining pipe MP#1 decreasing with the increase of the distance between the upper benches and the top surface of the mining pipe MP#1.

The sill pillar recovery process commences from simulation step S84 and ends at steps S94, S98, and S98 by scheme SBS, SS1, and SS2, respectively. Table 4 presents the average displacements at the upper bench levels of the analyzed locations induced by the sill pillar recovery and backfilling process. 

From Figure 9 and Table 4, the displacements of the analyzed locations at the upper bench levels caused by the process of sill pillar recovery conducted by the three sill pillar recovery schemes have a slight increase from upper level-1 (UL-1) to upper level-4 (UL-4). The maximum one is 0.303 mm at the upper level-4 (UL-4) from sill pillar recovery scheme SS2. The three sill pillar schemes are feasible because of the small displacements of the analyzed upper-level locations in the numerical model and monitoring prisms at the in-situ field. 

## 6. Assessment of the Stope Accesses Stability among Three-Pillar Recovery Schemes

In underground mining, stope accesses are excavated to connect the stopes and haulages, and stope accesses are used as paths to transport the minerals and other materials between the ground surface and underground working sites. Compared to other underground mining structures, stope accesses serve the longest time for the production in the mine. Therefore, the stability of the stope accesses has a significant effect on the production schedule. Thus, assessing the stability of the stope accesses is critical in assessing the feasibility of the sill pillar recovery.

**Figure 10:**
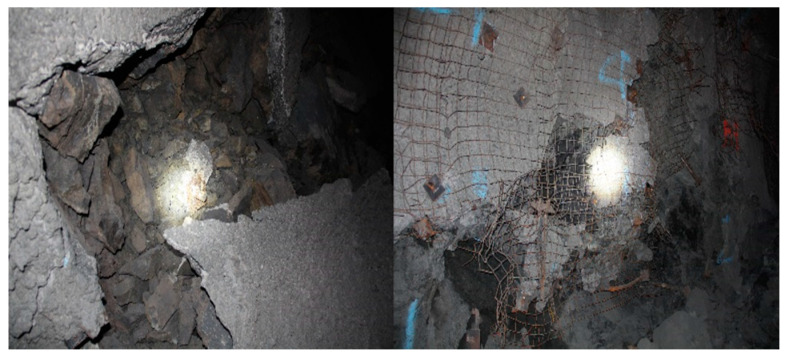
Failure cases near the stope access.

According to the studies done by Sepehri [30], Leveille [49,50], and Pu [51], kimberlite and granite in the hard rock mine have the potential for rockburst, which could cause severe damage to the roofs and sidewall surfaces in the stopes and crosscuts. In addition, some reported rockburst failure cases located near the stope accesses in this hard rock mine, as shown in Figure 10. Hence, the stability of the stope access is a key issue to be assessed.

### 6.1. Stress Condition in the Two Sill Pillars Pre-Sill-Pillar-Recovery

**Figure 11:**
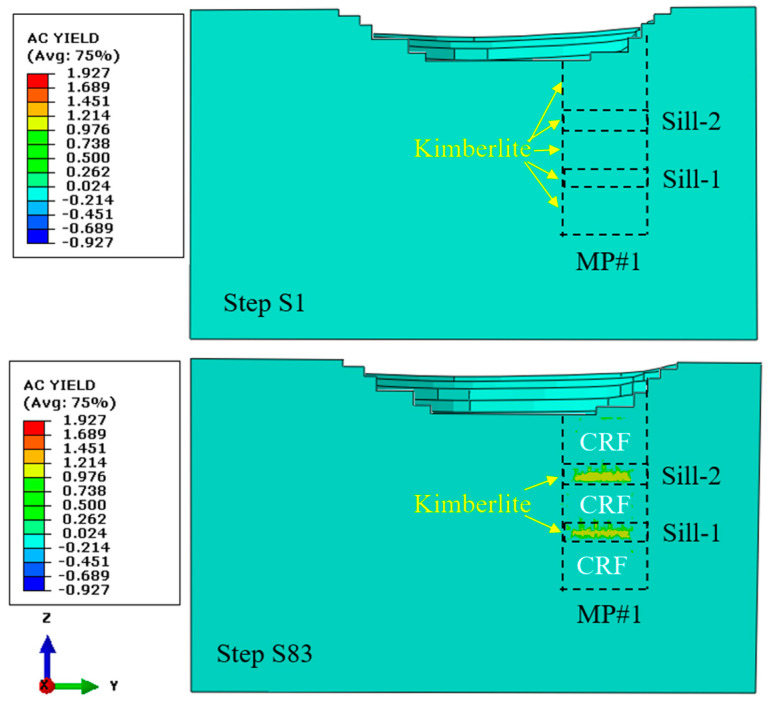
Yield zones in the MP#1 at step S1 and step S83.

In this hard rock mine, the reserved sill pillar consists of several mining stopes, and each stope can contain thousands of tons of minerals. Compared with the backfilled CRF in the mined voids, the unmined stopes in the sill pillar will provide more support than the backfilled CRF [39]. After completing the excavation and backfilling of the three mining blocks, the stress field changed and was redistributed. Due to the vast volume of the backfilled CRF, there are some yield zones in the unmined sill pillars. Figure 11 presents the yield zone in the two sill pillars at steps S1 and S83. In Figure 11, in the mining pipe MP#1, the yellow areas are the two sill pillars, and the other zones are backfilled CRF. 

### 6.2. Assessment of the Stope Access Stability among Three-Pillar Recovery Schemes

#### 6.2.1. Tangential Stress Criterion (Ts)

For a rockburst to occur, the rock mass must have the ability to store a considerable amount of strain energy which can be released violently at failure, and there must be an environment for stress concentration and energy accumulation [33,52,53]. Here, the tangential stress criterion was used to compare the stability assessment among the three sill pillar recovery schemes, SBS, SS1, and SS2. According to Wang and Park [53], the rockburst tendency can be evaluated using *Ts* criterion, as presented in Table 5.

**Figure 12:**
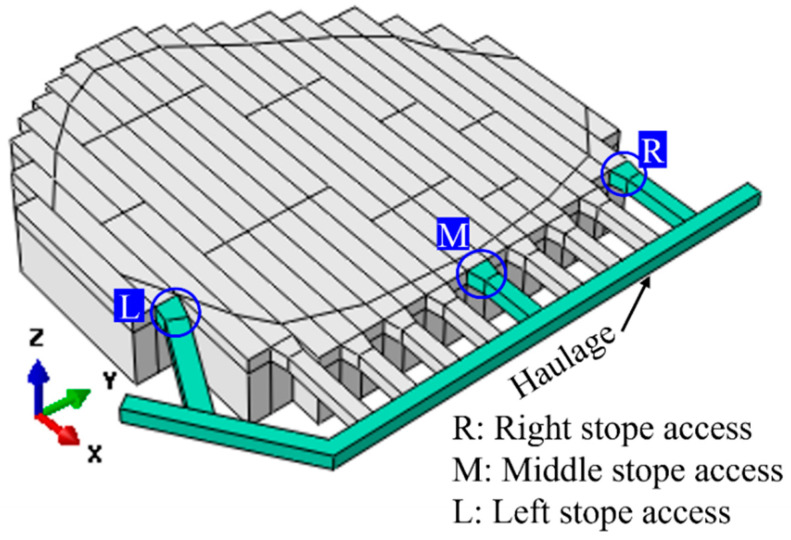
Analyzed stope access in sill pillar Sill-1.

In a two-dimensional study, with the influence of the backfilled CRF, in the process of sill pillar recovery, the two edges of the mining pipe have a higher possibility of instability in the overcuts and undercuts compared to the crosscuts in the middle area of the mining level [32,33]. The stope access from three representative locations of left-edge, middle line, and right-edge are analyzed to conduct the assessment, as shown in Figure 12. Both the left-edge stope access and right-edge stope access are close to the boundaries between the mining pipe MP#1 and the host rock granite. The stope access has a section size of 5 m × 5 m (height × width).

According to Sepehri [30] and Xu [32,33,39], the boundaries of the mining pipe MP#1 have higher tangential stress than the center of the mining pipe, and with the increase of the mining depth, the possibility of the potential rockburst is higher. Hence, the stope accesses to the undercuts of stopes in Sill-1 from the three locations are chosen to conduct the assessment.

**Figure 13:**
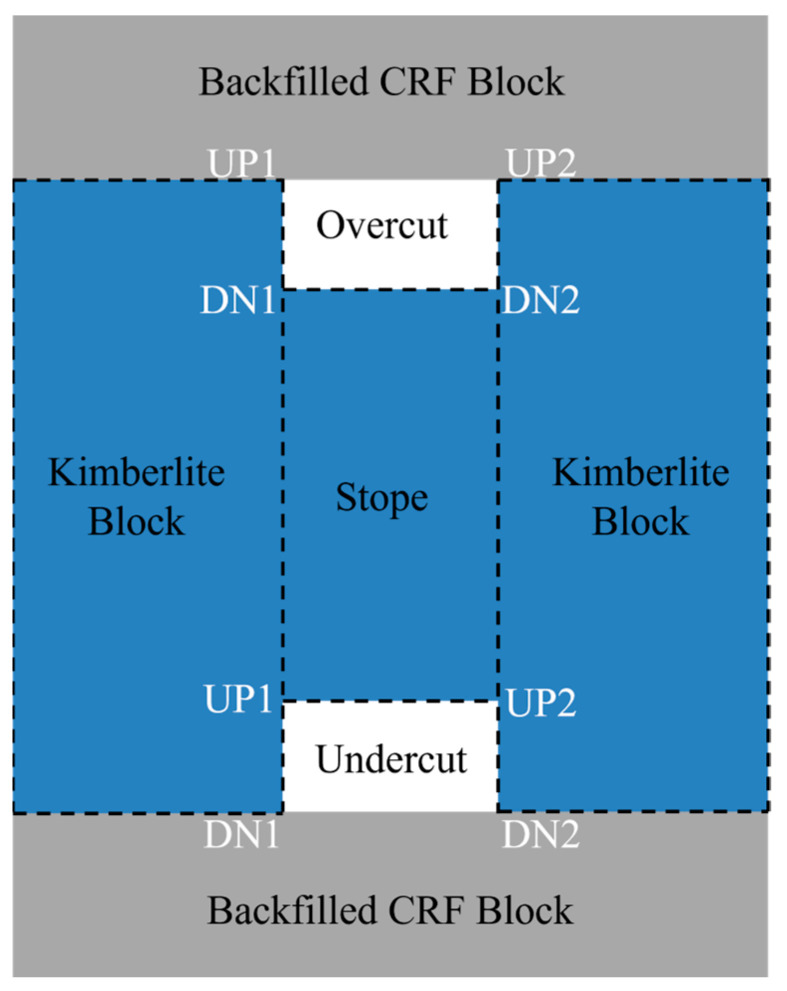
A profile of the chosen corners in the overcut and undercut accesses.

In underground rectangular openings, the four corners will easily generate the stress concentration effect, which may cause failures at these locations [33]. Four corners in the overcut and undercut are chosen to conduct the stability assessment comparison among the three sill pillar recovery schemes; UP1 is the left roof corner, UP2 is the right roof corner, DN1 is the left floor corner, and DN2 is the right floor corner, as shown in Figure 13.

All three sill pillar recovery schemes start at simulation step S84; the following figures start from simulation step S83, which is the last step of excavation and backfilling of the three mining blocks, and simulation step S84 is the first step to recover the sill pillars by three recovery schemes. The Ts values are compared among three sill pillar recovery schemes at the four corners in the stope access section at three stope locations. 

**Figure 14:**
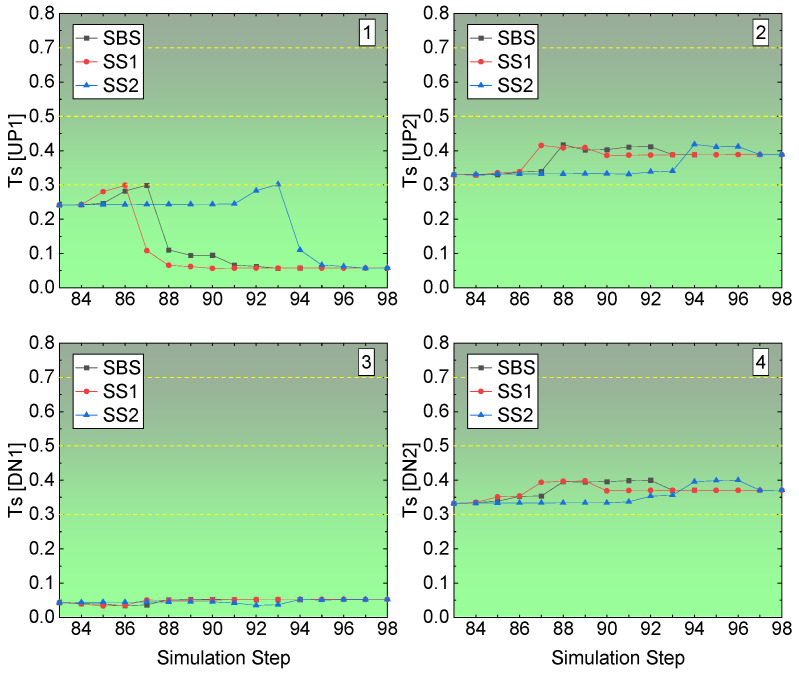
Ts value of the right-edge stope access to undercuts in Sill-1.

The Ts of the four corners at the undercut access in the right-edge stope in Sill-1 is shown in Figure 14. For the left roof corner UP1, the Ts value is less than 0.3, though at some step it reaches 0.3, and with the backfilling coming into effect at the flowing step, it goes down to a value under 0.1, then is safe for this corner based on the Ts value, and according to Table 5, there is no rockburst tendency. For the right roof corner UP2, from the beginning of the recovery, it is over 0.3, which means it is not stable as the left roof corner. Referring to Table 5, with a Ts value between 0.3 and 0.5, there is a rockburst tendency, though it is weak. For the left floor corner DN1, the Ts value is always under 0.1, which means there is no tendency of rockburst. For the right floor corner DN2, similar to the right roof corner, the value of Ts is between 0.3 and 0.5, presenting a weak tendency of rockburst. Comparing the Ts value at the four corners in the stope access to the undercut of the right edge in the Sill-1, the right side of the undercut access is more unstable than the left side. The right roof corner and floor corner present weak rockburst tendency, while the left side has no rockburst tendency.

**Figure 15:**
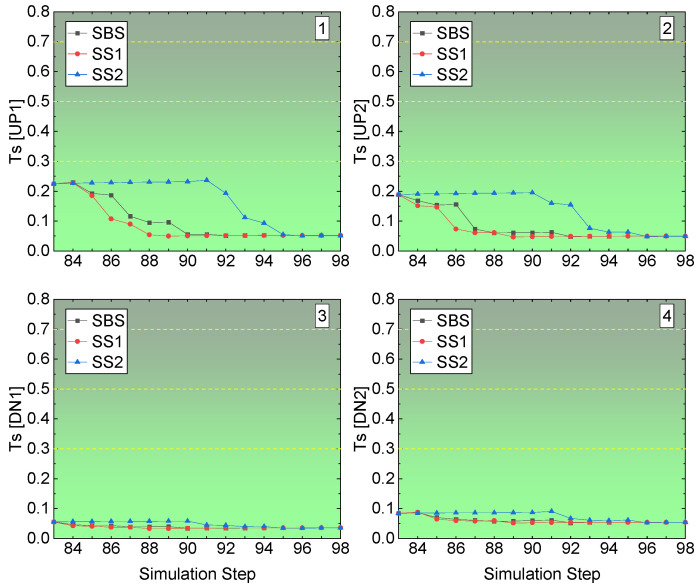
Ts value of the middle stope access to undercuts in Sill-1.

The rockburst tendency at the four corners in the middle stope undercut access in Sill-1 is shown in Figure 15. Unlike the right-edge stope access to the undercut, the four corners in the middle stope access to undercut show no rockburst tendency. Both the corners of the floor have a value of Ts less than 0.1, while the roof corners have a more considerable Ts value, though it is under 0.3. In Figure 13, the roof corners are in the unmined kimberlite rock mass, while the floor corners are in the backfilled CRF block. Although, according to the rock mechanics properties parameters in-put in the developed FE model in Table 1, the backfilled CRF has lower strength and elastic properties than the kimberlite block, when the backfilling finishes, the backfilled CRF could fail, but not in the type of rockburst.

**Figure 16:**
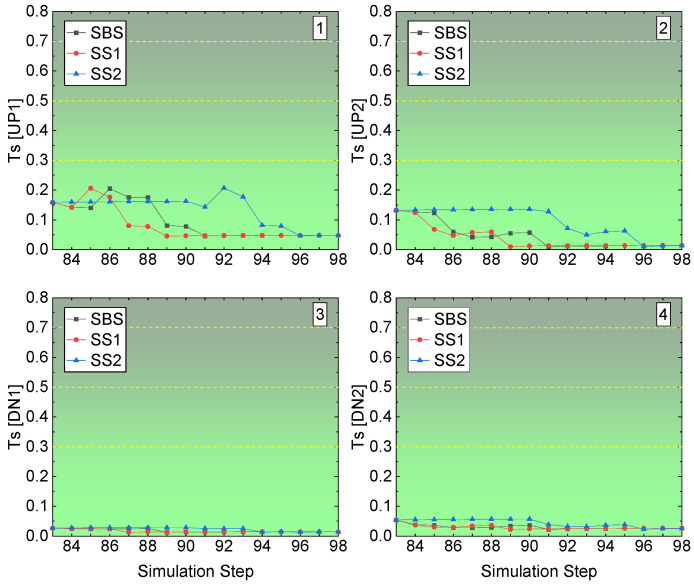
Ts value of the left-edge stope access to undercuts in Sill-1.

The rockburst tendency of the four corners in the left-edge stope access to the undercut is illustrated in Figure 16. Like the middle stope access to undercut, there is no rockburst tendency in the four corners. While compared with the floor corners, the roof corners have high values of Ts. The floor corners have a Ts value under 0.05 among the three sill pillar recovery schemes. For the roof corners, UP1 and UP2, though the three sill pillar recovery schemes cause a different value of Ts, the Ts is under 0.2, and after the completion of the recovery and backfilling of the two sill pillars, the Ts values of the three sill pillar recovery schemes reach the same final state of Ts.

Referring to Figure 12, in which the locations of the three analyzed stope accesses were marked, based on the results presented in Figure 14, Figure 15 and Figure 16, the right-edge stope access has a higher value of Ts, indicating the rockburst tendency at the right side of the right-edge stope access is higher than any other location in the other two stope accesses, the left-edge one and the middle one. 

#### 6.2.2. Energy-Based Burst Potential Index Criterion (BPI)

According to the studies of [30,49,50,51], the rockburst that occurred at this mine was classified as strainburst. Mitri [54] proposed the energy-based burst potential index (BPI) to better assess and predict the rockburst in the underground rock mass. Violent failure (rockburst) will occur when the energy stored in the rock mass exceeds the critical energy value (*e_c_*) [54,55,56,57,58]. The *e_c_* is the maximum capacity of the rock mass to store energy, and it can be obtained from the uniaxial compressive strength (UCS) test or the UCS hysteresis looping test curve with the following equation:

(1) $$e_{c}=\sigma_{c}^{2}/2E$$

where *σ_c_* is the UCS and *E* is Young’s modulus in the UCS test. It should be noted that estimating *e_c_* using Equation (1) is a conservative approach because the energy dissipated by fracturing and plastic deformations is neglected. Therefore, the BPI can be defined as

(2) $$BPI=ESR/e_{c}\times 100\%$$

where *ESR* is the energy storage rate (kJ/m^3^) in the rock mass, and *e_c_* is the critical elastic strain energy density (SED) (kJ/m^3^) of the rock. The larger the value of the BPI, the higher the probability of a rockburst occurring. According to Leveille [48], kimberlite’s average critical energy (*e_c_*) value in mining pipe MP#1 is 119.7 kJ/m^3^.

Based on the computed ESR from the FE analysis model, the burst potential index (BPI) of the four corners of stope accesses to the undercuts and overcuts in the Sill-1 and Sill-2 during the process of recovery of two sill pillars conducted by the three recovery schemes can be calculated, and the following figures present the results.

**Figure 17:**
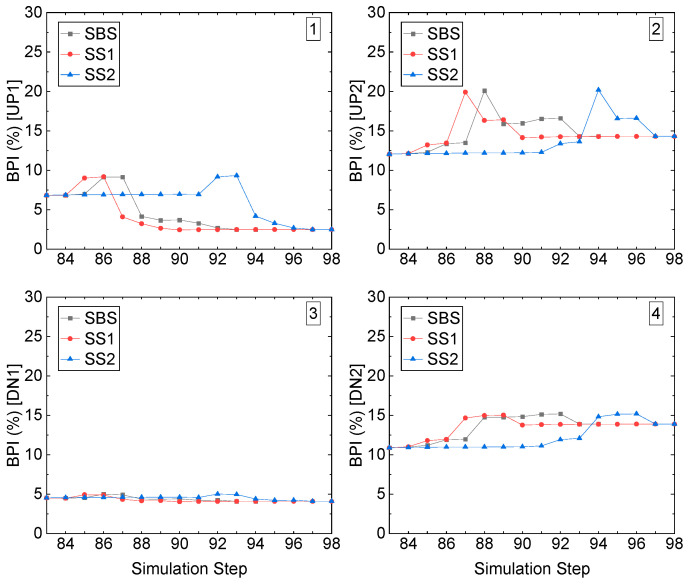
BPI value of the right-edge stope access to the undercut in Sill-1.

At the right-edge stope access, all four corners have a low value of BPI, as shown in Figure 17. For the left roof corner UP1, during the whole process of recovery, among the three recovery schemes, the BPIs are less than 10%, though with a slight increase caused by the excavation. On the other hand, for the right roof corner UP2, the BPIs see a swift increase caused by the excavation of the stope access at the specific simulation steps among the three recovery schemes.

Before the excavation occurs, the BPIs stay at a stable value of 12.5%; due to the excavation, it increases to 20%, after the backfilling, and then comes down to the initial value of 12.5%, which proves the backfilled CRF can effectively lower the BPI by providing the immediate support. The left floor corner DN1 has the lowest BPI among these four corners in the stope access, and it is 5%, keeping stable. For the right floor corner, with an increase of 4.5% from 10.5% to 15%, all three recovery schemes end at 14%.

**Figure 18:**
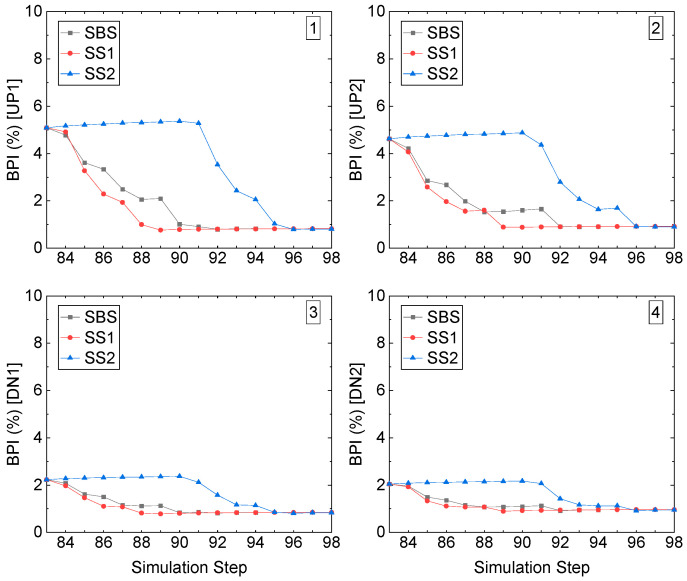
BPI value of the middle stope access to the undercut in Sill-1.

For the middle stope access to undercut in Sill-1, compared with the right-edge one, the BPIs are much smaller, as shown in Figure 18. For the two roof corners, UP1 and UP2, which have the highest BPI compared to the floor corners, the BPIs are not larger than 5%, and the excavation simulation step causes a decrease at both roof corners, as shown in the first and second pictures. On the two-floor corners, DN1 and DN2, as shown in the third and fourth pictures, the BPIs are even much smaller; both are less than 2%, though with the process of excavation, among the three recovery schemes, for recovery schemes SS2, before the excavation occurs, the BPI keeps stable as 2%.

**Figure 19:**
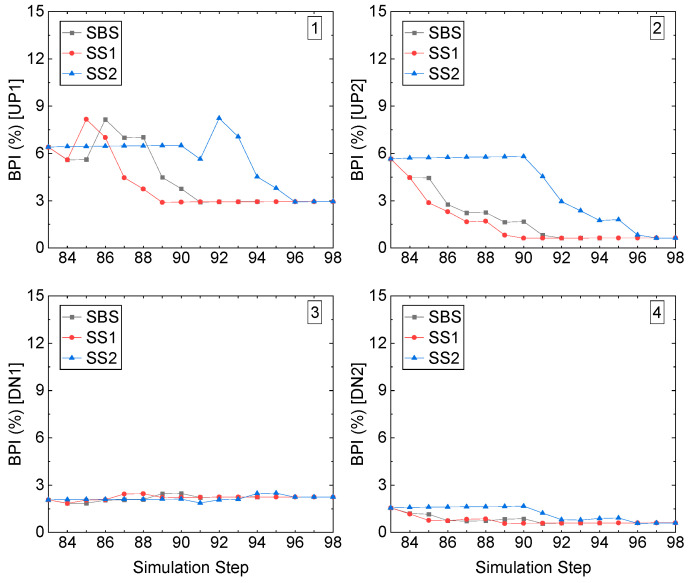
BPI value of the left-edge stope access to the undercut in Sill-1.

Figure 19 illustrates the BPI of the four corners in stope access to undercut in the left-edge stope in Sill-1. Like the right-edge stope access, the left roof corner UP1 increases, caused by the excavation, and the subsequent decrease, caused by the backfilled CRF, among the three recovery schemes. All the BPIs of the three recovery schemes are less than 9%, though the excavation caused the increase. The right roof corner, UP2, sees a constant decrease during the excavation and backfilling sill pillar recovery, and it is under 6% the whole process. For the two-floor corners, DN1 and DN2, the BPIs are under 3%, and for DN2, the BPI is even under 1.5%.

Similar to the tangential stress criterion (Ts) results, compared with the stope accesses to the undercuts in left edge-located stope and middle stope in Sill-1, the right-edge stope access has a higher value of BPI due to the contact boundary of kimberlite and granite. 

## 7. Discussion

In the previous studies [32,33], the authors used the two-dimensional (2D) models to analyze the displacement of the crosscuts to assess the stability of the stopes, and the displacement was the only factor in conducting the assessment. To make a more precise and comprehensive analysis and prediction, a full-sized elastoplastic three-dimensional (3D) model was developed in this paper by simulating the whole mining schedule process of stope excavation and backfilling. Different from other studies [1,2,3,4,5,6,7,12,14,16,17], in the presented research, a full-size 3D analysis model of the mine was constructed to make the predication and analysis more precise and accurate by simulating the whole process of excavation and backfilling of the stopes, and rockburst tendency as the key factor was considered, and three different sill pillar recovery schemes were proposed. 

The developed three-dimensional (3D) finite element (FE) analysis model was firstly calibrated and verified. Then, the sill pillar recovery simulation by three recovery schemes, SBS, SS1 and SS2, was conducted to investigate the feasibility of two sill pillars recovery. Finally, the displacement of the four monitoring prisms and four upper levels were chosen as indicators of the failure of the protective dike. 

According to the displacement results of the four upper bench levels, the three sill pillar recovery schemes had an extremely low possibility of causing failures of the four upper bench levels and the dike since the maximum displacements of the monitoring prism and the upper level was 2.99 mm (CRF-S) and 0.303 mm (UL-4), respectively.

The tangential stress criterion (Ts) and burst potential index criterion (BPI) were introduced and applied to assess the stability of the stope access to the two sill pillars during the process of the sill pillar recovery. The results of the sill pillar Sill-1 were presented since the mining depth of sill pillar Sill-1 was deeper. Though, at the access to the right-edge stope, weak rockburst tendency presented, advanced measures, such as stress-release, can be taken to reduce the damages caused by rockburst occurrence.

## 8. Conclusions

With an average relative error of 5.29% from the displacement comparison between the in situ recorded data and the FE model computed results, the developed numerical model was calibrated and proved to be capable of assessing the feasibility of the sill pillar recovery in the hard rock mine.

The maximum ground displacements caused by the recovery of the two sill pillars at the four monitoring prisms were 1.78 mm (280-10), 0.98 mm (280-12), 2.99 mm (CRF-S), and 2.07 mm (CRF-N), respectively. In addition, the displacements of the four upper bench levels caused by the sill pillar recovery were also presented, and the maximum displacement at each upper bench level was 0.18 mm (UL-1), 0.139 mm (UL-2), 0.294 mm (UL-3), and 0.303 mm (UL-4), respectively, which indicated that the protective dike would be stable during the sill pillar recovery.

During the process of sill pillar recovery, according to the tangential stress criterion (Ts) results, in Sill-1, the stope accesses to the right-edge stope had a weak rockburst tendency, while the accesses to the left-edge and middle stope did not tend rockburst. Similar to the Ts criterion scenarios, the results of BPI criterion showed that only the right-edge stope access in Sill-1 has a possible rockburst tendency at the right roof corner and the right floor corner. 

Considering the displacement of the monitoring prisms and the upper bench levels above mining pipe MP#1, and the stability of the stope accesses in the sill pillar Sill-1, all three sill pillar recovery schemes are feasible and rational. Therefore, any scheme can recover the two sill pillars. However, the recovery scheme SBS, starting the sill pillar recovery process from both sill pillars, is the optimum one among the three schemes. This is because the recovery scheme SBS simultaneously conducts the mining activities in both two sill pillars, which takes less time than the other two recovery schemes. Hence, recovering the two sill pillars by scheme SBS is more productive than the other schemes. In addition, recovering the two sill pillars by scheme SBS will reduce the cost of ventilation and other maintenance by taking less time in the recovery process, thus increasing the profit of the mine.

## Figures and Tables

**Table 1:** Properties applied in the numerical model.

Rock Mass	*γ* (MN/m^3^)	*C* (MPa)	*ϕ* (°)	*E* (GPa)	*ν*	*σ_c_* (MPa)
MP#1	0.024	4.7	28.1	19.6	0.24	79
CRF	0.022	1.2	35	2	0.3	1.5
Granite	0.026	9.3	45	24	0.3	130

**Table 2:** In situ displacement and the FE model computed displacement.

Monitoring Prism Location	FE Model (mm)	In-Situ Data (mm)	Relative Error (%)
Monitoring zone 1 (Ave-S01 & S02)	45.50	46.7	2.57
Monitoring zone 2 (Ave-N01 & N02)	36.38	39.55	8.01
Average relative error			5.29

**Table 3:** Sill pillar recovery induced displacement at monitoring zones.

Monitoring Zone	Displacement Caused by Recovery Scheme		
	SBS (mm)	SS1 (mm)	SS2 (mm)
280-10	1.77	1.73	1.78
280-12	0.97	0.86	0.98
CRF-S	2.99	2.89	2.97
CRF-N	2.07	2.00	2.06

**Table 4:** Sill pillar recovery induced displacement at upper levels.

Analyzed Location	Recovery Scheme		
	SBS (mm)	SS1 (mm)	SS2 (mm)
UL-1	0.168	0.147	0.180
UL-2	0.128	0.111	0.139
UL-3	0.284	0.294	0.294
UL-4	0.294	0.282	0.303

**Table 5:** Rockburst tendency prediction based on tangential stress criterion.

Tangential Stress Criterion (Ts)	Rockburst Tendency
Ts ≥ 0.7	Violent
0.5 ≤ Ts < 0.7	Strong
0.3 ≤ Ts < 0.5	Weak
Ts < 0.3	No rockburst
